# NEUROCOGNITIVE CORRELATES OF COMORBIDITIES IN OLDER ADULTS WITH CHRONIC PAIN AND NEGATIVE EMOTIONS

**DOI:** 10.1093/geroni/igae098.3320

**Published:** 2024-12-31

**Authors:** Irina Mindlis, Lisa Ravdin, M Carrington Reid, Dimitris Kiosses

**Affiliations:** Weill Cornell Medicine, New York, New York, United States; Weill Cornell Medicine, New York, New York, United States; Weill Cornell Medicine, Weill Cornell Medicine, New York, United States; Weill Cornell Medicine, New York, New York, United States

## Abstract

Chronic pain and depression are associated with poor cognition in older adults, yet little is known regarding the additional contribution of comorbidities to performance on specific neurocognitive domains in this population. We examined specific neurocognitive domain performance using baseline data from the Problem Adaptation Therapy for Pain Collaborative Care Program (PATH-Pain) trial, which tested a psychosocial intervention designed to improve emotion regulation in 100 adults ≥ 60 years with comorbid chronic pain and negative emotions (vs. usual care). Participants completed questionnaires on comorbidities (Charlson Comorbidity Index), depressive symptoms (Montgomery-Asberg Depression Rating Scale), pain intensity, and were remotely administered the Montreal Cognitive Assessment with no visual elements (MoCA-blind) and the Repeatable Battery for Neuropsychological Status. Regression assessed the relationship between specific neurocognitive domains and comorbidities, adjusting for depressive symptoms and pain intensity. In adjusted models, greater comorbidities remained associated with poorer immediate memory (b = -.352, p <.001) adjusting for pain intensity (b =.217, p =.040) and depressive symptoms (ns). After excluding participants with neurologic conditions, immediate memory remained associated with comorbidities (b = -.304, p =.019), but not pain intensity. Similar patterns emerged for attention, with greater comorbidities associated with poorer performance among all participants (b = -.266, p =.015), and for participants without neurological diagnoses (b = -.301, p =.020). No associations were found with language, delayed memory, abstraction, or orientation. Findings suggest difficulties with initial encoding and verbal learning; further research is needed to understand the potential impact on health behaviors and outcomes.

